# Extracurricular music and visual arts activities are related to academic performance improvement in school-aged children

**DOI:** 10.1038/s41539-023-00155-0

**Published:** 2023-03-29

**Authors:** Chiaki Ishiguro, Toru Ishihara, Noriteru Morita

**Affiliations:** 1 Department of Psychology, Kanazawa Institute of Technology, Nonoichi, Japan; 2 Graduate School of Human Development and Environment, Kobe University, Kobe, Japan; 3 Department of Sports Cultural Studies, Hokkaido University of Education, Iwamizawa, Japan

**Keywords:** Human behaviour, Human behaviour

## Abstract

The present longitudinal study examined whether extracurricular activities in the arts and corresponding scores in art classes have a positive association with general academic performance. Data were collected from 488 seventh-grade children (259 boys and 229 girls) for over two years. Information regarding their participation in extracurricular activities in music and visual arts, grade points in general academic performance (i.e., Japanese, Social Studies, Mathematics, Science, and English), music, and arts were obtained at the end of the seventh and ninth grades. Structural equation modeling revealed that participation in extracurricular activities in both music and visual arts was positively associated with improvements in general academic performance from the seventh and ninth grades, and these associations were related to changes in music and visual arts scores. This finding suggests that arts education can contribute to improving general academic performance; however, the current study shows correlational relationships. Future research should examine the causal relationship between art involvement and academic performance by controlling for other factors (e.g., IQ, motivation, etc.).

## Introduction

Arts education is believed to cultivate creativity and innovative skills, in addition to knowledge and skills that are specific to the arts. Many children have the option to participate in music and visual arts as extracurricular activities, which are organized, structured, involved, and supervised by adults outside school^1^, besides the art classes that are a part of formal education.

In Japan, all students take 35 to 45 50-min art classes each year, including music and visual arts. In addition, they can choose to participate in extracurricular activities that are provided after school and supervised by teachers. A national survey by the Japan Sports Agency (2018) revealed that almost all schools provide extracurricular activities, divided into sports clubs (e.g., tennis, basketball, and baseball) and cultural clubs (e.g., brass band, choir, and visual arts)^2^. In cultural clubs, music (such as choir and brass bands) and visual arts are the two most popular activities. Further, they reported that 71% of junior high school students participated in the sports club and 19% in the cultural club. In Japan, most club activities for junior high school students are conducted almost every weekday. Among the cultural clubs, brass band (9.8%) and visual arts club (4.5%) are the most popular. Approximately 75% of all junior high students in cultural clubs reported that they participated in club activities for more than four days a week, and 79% of the cultural club members spend 1–3 h each weekday on their club activities^2^. These varied types of arts education have been studied to understand their impact on academic performance.

Music education is one of the most studied fields in art education. Several studies have suggested a positive association between music participation and academic performance^3–5^. Wetter et al.^5^ showed a significant positive association between extracurricular music lessons and academic outcomes in 120 children. Furthermore, Southgate and Roscigno^6^ revealed that music involvement both inside and outside the school was associated with children’s and adolescents’ academic achievements (mathematics and reading), even if socioeconomic status and family background were controlled on a nationally representative data source in the US. Such results are replicated in a more recent survey on 11–12-year-olds in the US^7^. Although a few correlational studies indicate no significant relationship between music education and academic performance (for a review, see ref. ^8^), quasi-experimental findings suggest that IQ scores, which are related to academic performance, are higher in musically trained groups^9–12^.

The relationship between visual arts education and academic performance remains controversial. Vaughn and Winner^13^ reported that students who selected visual art courses as their secondary subject in high school achieved higher SAT scores, which is an exam taken for admission to US colleges and universities. Other studies investigated the popular visual art class–Visual Thinking Strategies (VTS)–and reported that VTS participants showed higher scores and performance in standard academic tests and critical thinking, respectively^14–16^. Although these studies demonstrated that students who were involved in visual art courses achieved higher grades, they have been criticized for being correlational studies and for not controlling baseline intellectual score and socioeconomic status^8^.

Although research in both music and visual arts education indicates a positive association between arts education and academic performance, there remains the following question: how can the skills developed in music and visual arts education transfer to academic performance? Psychologists have defined the transfer effect as the phenomenon in which learning in one domain is generalized to another domain^17^ or preparation to learn in another domain^18^. Based on this definition, Winner et al.^8^ considered the impact of arts education on non-artistic outcomes (general academic performance such as in mathematics, reading, and science) as a transfer effect of arts education. Furthermore, they claimed that arts education contributes to the activation of brain areas related to cognitive skills, social skills, motivation, and attitudes involved in non-art learning and emphasized the significance of examining mediating factors in the transfer^8^. According to these assumptions, they summarized their hypotheses on the transfer effect of arts education on academic outcomes in two ways. The first hypothesis posited a simple transfer of arts education; they assumed that arts instruction can lead to improvement in academic performance, even if the impact may be weaker than that of direct training in the academic domain. The second hypothesis posited that artistic skills are mediating factors in the transfer such that arts instruction can lead to the improvement of artistic skills and result in achieving certain academic outcomes (with a weaker impact than direct training in artistic skills). Although they also proposed additional hypotheses about the transfer effect of integrated arts education such as STEAM education, and about the transfer effect in real life situations other than the academic domain, these are not detailed here. Previous research has examined the first hypothesis, and results remain inconsistent. Thus, the current study aimed to examine the first and second hypotheses, that is: (1) whether music and visual arts education is associated with general academic performance; (2) whether the effect of music and visual arts education has an indirect effect on academic performance that is mediated by music and visual art performance (i.e., scores).

**Fig. 1:**
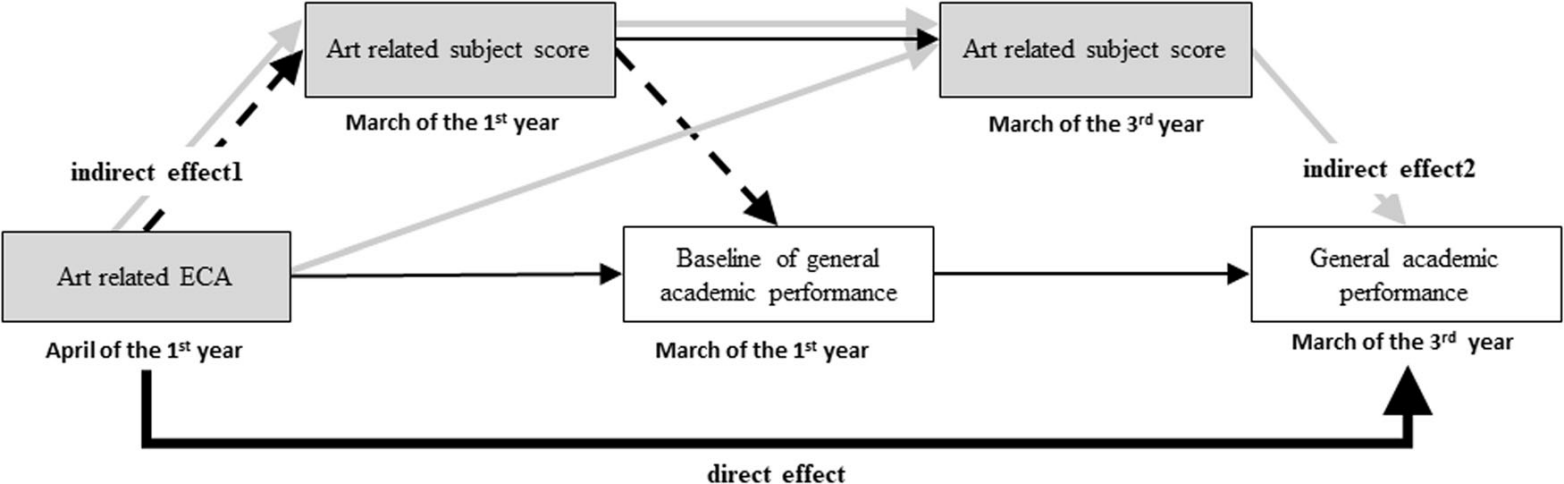
The hypothesis model in the current study. Shaded boxes show students’ art-related activity and grade scores and white boxes show the general ACGP. Bold line shows the direct effect of art-related ECA on general ACGP in the third year. Dotted line shows the indirect effect (indirect 1) of art-related ECA via art-related score in the first year on general ACGP in the first year. Gray line shows the indirect effect (indirect 2) of art-related ECA via art-related score in the first and third years on general ACGP in the third year. ECA means extracurricular activity, ACGP means general academic score which is calculated by five major subjects GP.

To test these hypotheses, a 2-year longitudinal survey was conducted, which focused on art involvement as extracurricular activities in junior high school students. Japanese children are required to study for six years in elementary school from the age of six (first through sixth grades) and three years in junior high school from the age of 12 (seventh through ninth grades). In junior high school, children study five major subjects (Japanese, mathematics, social studies, science, and English) and four secondary subjects (music, visual art, health and physical education, and technology and home economics), and can opt for art education as an extracurricular activity. Given that all children in Japan take music and visual art classes in junior high school as secondary subjects and can also increase involvement in them as extracurricular activities, data collection was possible. In addition, in the current study, we examined the five main subjects in the Japanese standard curriculum as general academic performance. Thus, the present survey measured art-related scores and general academic performance of Japanese junior high school students twice (in the first and third years), which made it possible to examine the association between improvement in arts subject scores and academic performance over two years. While most countries begin the school year in September, the Japanese school year starts in April. Therefore, junior high school students in Japan typically start their extracurricular activities in late April in the first year of junior high school (i.e., seventh grade), after the entrance ceremony and guidance. Children’s academic attainment from April to March in the next year is integrated at the end of each school year. This two-year longitudinal study allowed us to examine four aspects of the impact of extracurricular arts education (Fig. 1). The first is the direct influence of arts club participation on academic performance in the third year. The second is the indirect effect of arts club on academic performance via grades in each arts course. This can be divided into indirect effects in the first year (indirect 1) and third year (indirect 2). The indirect 1 can be examined using a cross-sectional survey framework to demonstrate the relationship between arts club, arts subject grades, and academic performance in the first year. In contrast, the indirect 2 comprises the association between arts involvement and children’s growth in art scores in the third year and academic performance for two years, which can be further divided into two categories: (1) the effect mediated by the third-year art-related scores from the extracurricular activity and (2) the effect mediated by the first and third years art-related scores from the extracurricular activity. Finally, these effects on academic performance in the third year will be integrated into total effects, which will allow us to determine the impact of extracurricular arts education on academic performance. Finally, this analytic procedure can demonstrate the transfer effects of arts education (Hypothesis 1) by examining the overall effect, and the mechanism of the transfer effect (Hypothesis 2) by examining the indirect effects of the first and second years (the second year is particularly important in examining the longitudinal effects). This study assumed these hypotheses by considering the following variables as control variables. First, this study set music and visual art scores in the first year as the baseline control variable to examine the impact of two-year change of music and visual art scores on academic performance. Second, considering previous research on the development of adolescents’ academic performance^19,20^, we set demographic variables such as sex, socioeconomic variables (i.e., household income and maternal education), and learning habits as the control variables. Table 1 shows a list of the definitions of terms used in the analyses. Further, previous research indicated that adolescents improve their academic performance by participating in sports or moderate-to-vigorous physical activities^21,22^ and there can be the influence of cultural extracurricular activities other than art and music, “other cultural club”. Therefore, we included these factors in the further analyses.

**Table 1:** Definition of the terms in this study.

Term	Definition
ECA	Extracurricular activity
GP	Final grade point in a subject summarized at the end of a school year
MUGP	Music GP
VAGP	Visual Arts GP
ACGP	General academic score which is calculated by five major subjects GP
Learning habits	Latent score which is calculated by the duration of learning after school on weekdays and weekends

## Results

Table 2 shows the demographic characteristics of the participating children and the descriptive statistics of ACGP, MUGP, and VAGP.

**Table 2:** Characteristics of the participating children.

Variables	ECA visual art		ECA music		Children who do not belong to ECA music or visual arts		All	
*N* (Boys/Girls)	21 (4/17)		53 (7/46)		414 (248/166)		488 (259/229)	
Time1 (first year)								
ACGP	17.68	(3.68)	18.52	(3.74)	17.97	(4.03)	17.96	(3.98)
MUGP	4.09	(0.74)	3.52	(0.81)	3.58	(0.73)	3.63	(0.75)
VAGP	3.87	(0.79)	4.19	(0.93)	3.61	(0.86)	3.66	(0.87)
Household income								
Less than 2,000,000 yen	0	(0)	2	(4)	23	(6)	25	(5)
2,000,000 yen to 4,000,000 yen	5	(24)	8	(15)	72	(17)	85	(17)
4,000,000 yen to 6,000,000 yen	5	(24)	16	(30)	151	(36)	172	(35)
6,000,000 yen to 8,000,000 yen	6	(29)	21	(40)	123	(30)	150	(31)
More than 8,000,000 yen	5	(24)	6	(11)	45	(11)	56	(11)
Maternal education								
Junior high school	1	(5)	1	(2)	8	(2)	10	(2)
High school	7	(33)	32	(60)	225	(54)	264	(54)
Vocational school	6	(29)	9	(17)	83	(20)	98	(20)
Junior college	6	(29)	6	(11)	72	(17)	84	(17)
Undergraduate studies	1	(5)	5	(9)	24	(6)	30	(6)
Graduate studies	0	(0)	0	(0)	2	(1)	2	(0)
Learning time in weekday								
<30 min	3	(14)	11	(21)	51	(13)	65	(14)
30 min–1 h	1	(5)	10	(19)	74	(18)	85	(18)
1–2 h	10	(48)	21	(40)	156	(38)	187	(39)
2–3 h	6	(29)	6	(12)	96	(24)	108	(22)
Over 3 h	1	(5)	4	(8)	30	(7)	35	(7)
Learning time in weekend								
<30 min	5	(25)	10	(19)	68	(17)	83	(17)
30 min–1 h	2	(10)	10	(19)	60	(15)	72	(15)
1–2 h	8	(40)	19	(36)	139	(34)	166	(34)
2–3 h	5	(25)	12	(23)	97	(24)	114	(24)
Over 3 h	0	(0)	2	(4)	47	(11)	49	(10)
Time2 (third year)								
ACGP	17.57	(4.49)	18.95	(3.89)	17.72	(4.62)	17.76	(4.57)
MUGP	4.57	(0.74)	3.86	(0.73)	3.75	(0.88)	3.84	(0.89)
VAGP	3.81	(0.86)	4.38	(0.67)	3.64	(0.82)	3.69	(0.83)
Learning time in weekday								
<30 min	0	(0)	4	(10)	24	(8)	28	(8)
30 min–1 h	1	(7)	2	(5)	26	(8)	29	(8)
1–2 h	4	(27)	9	(23)	81	(26)	94	(26)
2–3 h	7	(47)	11	(28)	93	(30)	111	(30)
Over 3 h	3	(20)	13	(33)	87	(28)	103	(28)
Learning time in weekend								
<30 min	0	(0)	4	(10)	29	(9)	33	(9)
30 min–1 h	1	(7)	2	(5)	19	(6)	22	(6)
1–2 h	2	(13)	7	(18)	65	(21)	74	(20)
2–3 h	6	(40)	7	(18)	66	(21)	79	(22)
Over 3 h	6	(40)	19	(49)	132	(42)	157	(43)

Data are presented as the mean ± SD. Household income and maternal education were calculated for the complete data (*N* = 488).

*ECA* extracurricular activity, *GP* final grade point in a subject summarized at the end of a school year, *MUGP* music GP, *VAGP* visual arts GP, *ACGP* general academic score which is calculated by five major subjects GP.

### Structural equation modeling

**Fig. 2:**
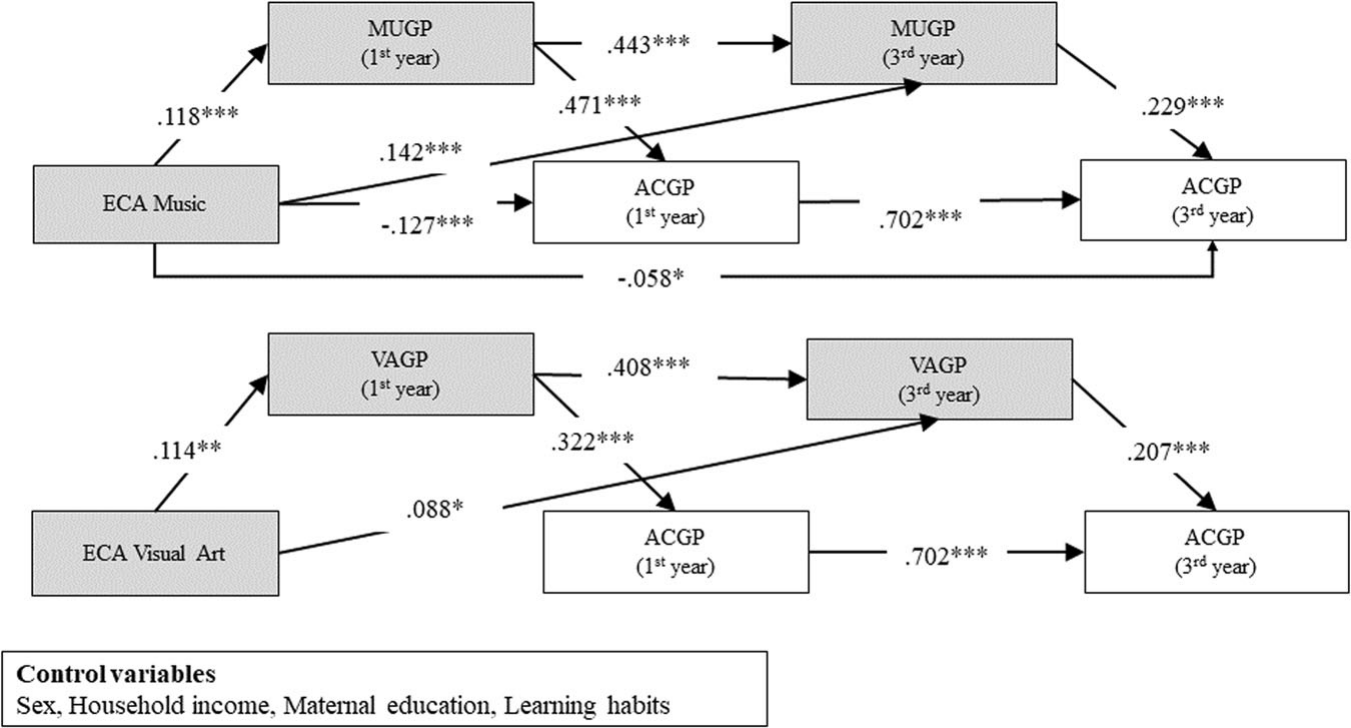
Direct and indirect pathways from extracurricular activity in music and visual arts to academic performance, mediated by music and visual arts scores with SES controls. Note: ECA is extracurricular activity, GP is final grade point in a subject summarized at the end of a school year, MUGP is music GP, VAGP is visual arts GP, and ACGP is general academic score which is calculated by five major subjects GP. Structural equation modeling *N* = 488, *χ*^2^ (df = 163) = 485.92 (*p* < 0.001), CFI = 0.95, GFI = 0.99, AGFI = 0.98, RMSEA = 0.06; dotted paths denote non-significant effects, while solid-line paths denote significant effects. Values are presented as standardized *β*(b*), after controlling for sex, household income, maternal education and learning habits.

In the following, ECA means extracurricular activity, and GP means final grade point in a subject summarized at the end of a school year. Further, the music GP, visual arts GP, and general academic performance, which was calculated by five major subjects GP, were abbreviated to MUGP (music GP), VAGP (visual arts GP), and ACGP (academic GP), respectively. Therefore, the structural equation model (SEM) showed an adequate fit (Fig. 2: *χ*^2^ (163, *N* = 488) = 485.92, *p* < 0.001, CFI = 0.95, GFI = 0.99, AGFI = 0.98, RMSEA = 0.06, 90% CI = 0.06–0.07, *p* < 0.001). Table 3 presents the direct, indirect, and total effects of the variables on the MUGP in ACGP in third year. With respect to music, although the indirect effect via MUGP in the first year on the ACGP in the first year was positive (indirect 1: b* = 0.055, *p* = 0.004), the direct effect of ECA music was negative (direct effect: b* = −0.058, *p* = 0.022), and the total effect was not positive (b* = −0.072, *p* = 0.071). The effect of ECA Music on MUGP in the third year was significantly positive (b* = 0.194, *p* = 0.000) and the indirect effects on the ACGP in the third year via MUGP in the first and third years were significantly positive (indirect 2: b* = 0.044, *p* = 0.000). However, the total effect remained insignificant.

**Table 3:** Standardized direct, indirect, and total effects for all variables in the model.

	Estimate	SE	*Z*	*P*	Lower	Upper	Stad.all
Music							
Direct	−0.126	0.055	−2.297	0.022	−0.234	−0.019	−0.058
Indirect on ACGP in first year via MUGP in first year (indirect 1)	0.109	0.038	2.878	0.004	0.035	0.183	0.055
Total on ACGP in first year from ECA	−0.141	0.078	−1.803	0.071	−0.294	0.012	−0.072
Total on MUGP in third year via MUGP in first year	0.548	0.116	4.707	0.000	0.320	0.776	0.194
Indirect on ACGP in third year via MUGP in third year (indirect 2)	0.097	0.025	3.897	0.000	0.048	0.145	0.044
Total on ACGP in third year from ECA	−0.139	0.084	−1.665	0.096	−0.303	0.025	−0.064
Visual arts							
Direct	0.059	0.080	0.742	0.458	−0.097	0.216	0.018
Indirect on ACGP in first year via VAGPin first year (indirect 1)	0.111	0.042	2.674	0.008	0.030	0.193	0.037
Total on ACGP in first year from ECA	0.080	0.111	0.728	0.467	−0.136	0.297	0.027
Total on VAGP in third year via VAGP in first year	0.545	0.168	3.248	0.001	0.216	0.874	0.135
Indirect on ACGP in third year via VAGP in third year (indirect 2)	0.093	0.032	2.932	0.003	0.031	0.155	0.028
Total on ACGP in third year from ECA	0.215	0.119	1.805	0.071	−0.019	0.448	0.064

Indirect 1 means the indirect effect of art-related ECA on ACGP in the first year mediated by art-related score (first year) and indirect 2 means the indirect effect of art-related ECA on AP in the third year mediated by art-related score (first and third years). Total effect means the sum of direct effects and indirect effects that lead to ACGP in first and third years.

*ECA* extracurricular activity, *GP* final grade point in a subject summarized at the end of a school year, *MUGP* music GP, VAGP: visual arts GP, *ACGP* general academic score which is calculated by five major subjects GP.

**Fig. 3:**
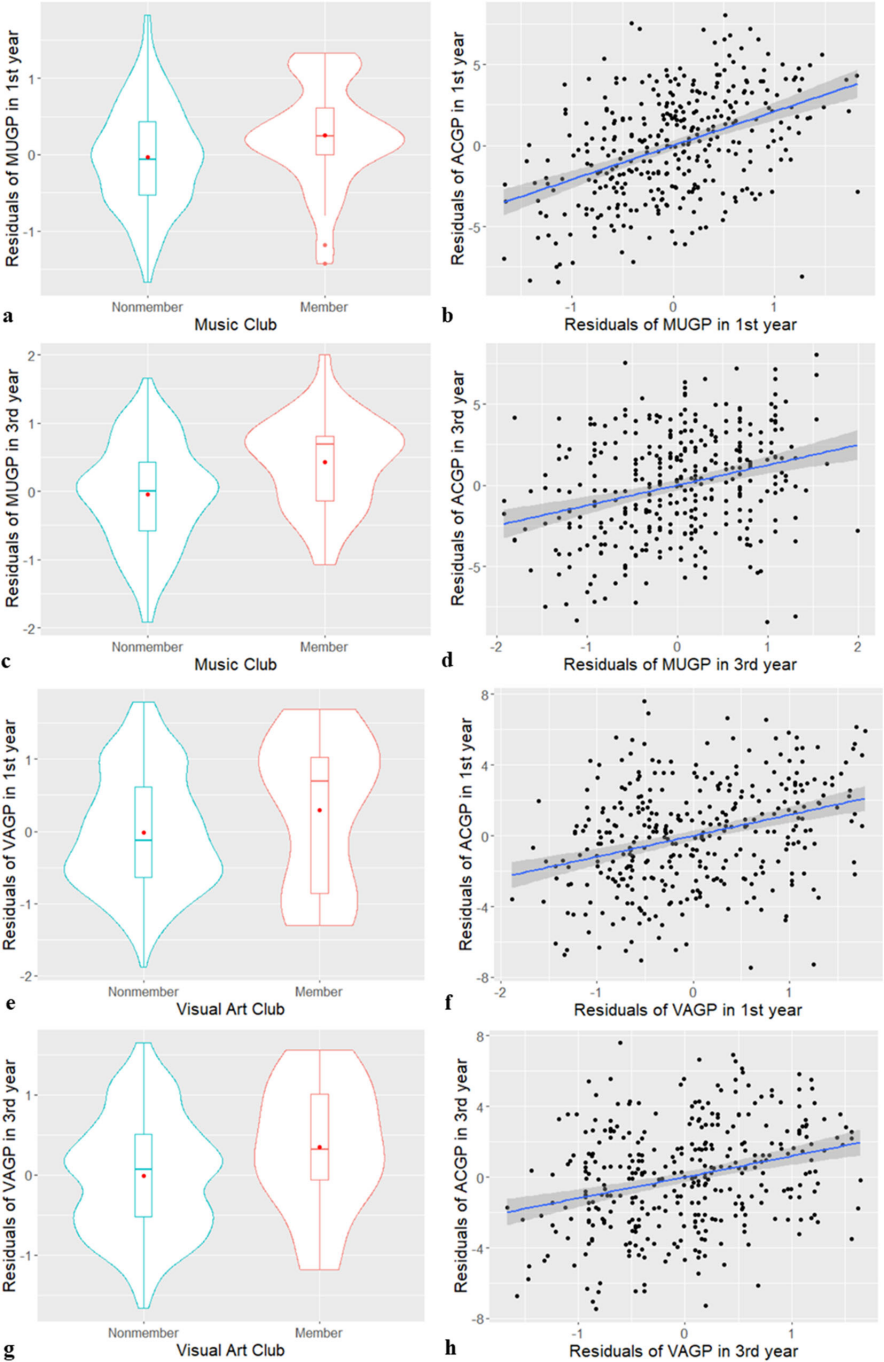
The indirect effects of art-related clubs on AP in the first and third years via art-related GP in the first and third years. Note. ECA is extracurricular activity, GP is final grade point in a subject summarized at the end of a school year, MUGP is music GP, VAGP is visual arts GP and ACGP is general academic score which is calculated by five major subjects GP. Panels **a**, **c**, **e**, and **g** show comparisons of the grade points of artistic subjects between joining and not joining ECA clubs. The difference means an influence of joining or not joining ECA clubs, which is equal to the former parts of the indirect transferring effects of ECA activities. Panels **b**, **d**, **f**, and **h** indicate relationships between the grade points of artistic subjects and general academic performance from the main subjects. The associations mean the later parts of the indirect transferring effects of ECA activities. **a** MUGP in joining or not joining music clubs in the first year; **b** Scatter plots for MUGP and general ACGP in the first year; **c** MUGP in joining or not joining music clubs in the third year; **d** Scatter plots for MUGP and general ACGP in the third year; **e** VAGP in joining or not joining art clubs in the first year; **f** Scatter plots for VAGP and general ACGP in the first year; **g** VAGP in joining or not joining music clubs in the third year; **h** Scatter plots for VAGP and general ACGP in the third year.

Regarding visual arts, although the indirect effect via VAGP in the first year on the ACGP in the first year was positive (indirect 1: b* = 0.037, *p* = 0.008), the direct effect of ECA visual arts was not significant, and the total effect was not significant (b* = 0.027, *p* = 0.467). The effect of ECA Visual Arts on VAGP in the third year was significantly positive (b* = 0.135, *p* = 0.001) and the indirect effect on the ACGP in the third year via VAGP in the first and third years were significantly positive (indirect 2: b* = 0.028, *p* = 0.003). However, the total effect was marginally insignificant (b* = 0.064, *p* = 0.071). The results of this analysis are summarized in Supplementary Table 1. Figure 3 shows the paths from art-related ECA to ACGP in the first and third years via each art-related subject GP. Note that MUGP, VAGP and ACGP scores show the residuals after adjusting the effect of the control variables. Violin plots show that art-related ECA participants scored higher in each art-related subject GPs in first and third years. In addition, the scatter plots indicated that art-related GPs were associated with ACGP in the first and third years in both art-related subjects. While we assumed the one-directional model that art-related ECAs transfer ACGP via art-related GP, it can be possible to assume the inversed effect of ACGP on art-related GP. Therefore, we checked the inversed model that assumes art-related ECA influences on art-related GPs via ACGP in the first and third years (this is a model with ACGP and GP positions swapped from the Fig. 1 model). Therefore, there were no indirect relationships; that is, there was no significant mediating role of ACGP between art-related ECA and GPs (Supplementary Fig. 1).

It is possible that the changes in the academic performance of children who participated in sports extracurricular activities affected the results. Therefore, we also tested the hypothetical model in the participants who do not belong to extracurricular sports. We excluded the data of those who were engaged in extracurricular activities related to exercise from the data set before excluding the missing SES (*N* = 700). Finally, the dataset for the second analysis was *N* = 147 (male, *n* = 41; female, *n* = 106), excluding missing data of SES (*N* = 56). We then applied the arts hypothesis and structural equation modeling to the sports excluding data. Therefore, the structural equation model showed an adequate fit (*χ*^2^ (175, *N* = 147) = 299.08, *p* < 0.001, CFI = 0.94, GFI = 0.98, AGFI = 0.97, RMSEA = 0.07, 90% CI = 0.06–0.08, *p* = 0.011). Supplementary Table 2 presents the direct, indirect, and total effect of variables on GP in general AP in the third year. With respect to music, although the direct effect of ECA music was insignificant, the indirect 1 and 2 were still significantly positive (indirect 1: b* = 0.102, *p* = 0.008; indirect 2: b* = 0.105, *p* = 0.001), and the total effect was insignificant (b* = 0.055, *p* = 0.466). However, with respect to visual arts, although ECA visual arts had an insignificant indirect effect 1 (b* = 0.039, *p* = 0.188), the indirect effect 2 was insignificant but positive (indirect 2: b* = 0.026, *p* = 0.096) and the total effect was significantly positive (b* = 0.168, *p* = 0.016) (see detailed results in Supplementary Table 3). The results of the indirect effects in ECA music and visual arts were consistent with the first analysis on a sample (*N* = 488) including all children who participate in ECA sports, music, visual arts, or other cultural activities as well as those who do not participate. However, the total effect of ECA visual arts was significant in the second analysis to compare children participating in extracurricular music and visual arts with the other children (participants of other extracurricular cultural activities and those who do not participate in any extracurriculars).

## Discussion

The current study examined the transfer effect of arts education in extracurricular activities on academic performance, that is, whether participation in the arts club has an impact on non-artistic outcomes. Further, the mechanism of the transfer effect was specified by assuming the mediating factor of improvements of artistic skills and knowledge. For these purposes, the current study investigated the direct and indirect effects of extracurricular activities in music and visual arts on each art subject score and general academic performance. Although the total and direct effects were different between music and visual arts, both extracurricular activities of music and visual arts were positively associated with changes in academic performance when they were mediated by each subject’s score change in the first and third years, which supported the second hypothesis of Winner et al.^8^. That is, children participating in music or visual arts clubs scored higher in their general academic performance, and the change was mediated by the increase in their music and visual arts scores. In addition, this relationship was not diminished even after controlling for the socioeconomic status factor.

This study illustrated the specific relationship between arts involvement and academic performance. Considerable attention has been paid to extracurricular sports activities (e.g., swimming, hockey, soccer, martial arts, etc.), and there has been a positive association between cognitive and social development and mental health^21,23–28^. Similarly, involvement in arts (e.g., music and visual arts) had a positive association with general academic performance. Considering that many countries have reduced the instruction time for music and visual arts subjects in the past decade^8^, this finding is significant for arts education. It is necessary to review and acknowledge the benefits of arts education.

Previous research has indicated a positive correlation between after-school music involvement and academic performance^4,5,9,29^. However, the current study indicated that the direct effect of after-school music involvement on academic performance was significantly negative when controlling for the SES and music score. Considering that there was a significant and positive indirect effect via music score in the third year and the total effect was insignificant and negative, it can be said that the direct effect decreased the transfer effect. The negative direct effect of extracurricular music on academic performance in the first (b* = −0.127) and third (b* = −0.058) years was larger than that of the indirect positive effects via music score in the first (indirect 1: b* = 0.055) and third (indirect 2: b* = 0.044) years. As a result, no significant total effects were obtained for the first (b* = −0.072) and third (b* = −0.064) years. Why did music club involvement have a negative impact on academic performance? Music clubs such as chorus and brass bands are reported to be the most frequent of cultural clubs;^2^ such clubs require students to practice extensively for instrumental performance and acquisition of choral skills. Therefore, the burden on students may have a negative impact on their learning of major subjects for the first year after joining a club. However, after about two years, the students become accustomed to the activities and acquire sufficient skills. Such music learning in club activities may begin to have a positive impact on their academics over time. These results support that the positive effect of after school music involvement with general academic performance in previous studies was explained not by the involvement, but by the outcome of music learning. According to Winner et al.^8^, music learning results in neurological, cognitive, social, and motivational activation, which are areas other than music. A recent study showed that a 30-min intervention of interactively playing music on a musical instrument improved children’s control over attention more than playing video games for the same amount of time^30^. Considering this recent finding, the experience in a music club (i.e., a brass band or choir) may have a similar impact on adolescents, resulting in higher academic performance.

The transfer effect of after-school music involvement and education on general academic performance is an important finding in music education research. Although the size of the transfer effect is quite small, it is surprising that the improvement of general academic performance from first to third year was explained by the change of the music score and extracurricular activities involved in it. Interestingly, the total effect from extracurricular music to general academic performance in the third year was not significant; however, the indirect effects from extracurricular music to general academic performance in the third year via music score were positively significant. This result implies that participation in music activities itself is not associated with the general academic performance; that is, music club children did not achieve higher scores in their first year of junior high school. However, they could improve their academic performance, and this change was mediated by the change of their music skills and knowledge. The current findings suggest that music learning for more than a year enables the transfer effect on academic performance. Overall, these results support the claim that the impact of music lessons is small but long-lasting^4,31^.

This study showed a positive association between after-school visual arts involvement and academic performance even after controlling for socioeconomic status. Past research on visual arts education has been criticized for using a correlational study design with a one-time survey without controlling for related variables such as baseline intellectual skills and socioeconomic status^29,32^. Contrary to the results of music extracurricular activities, visual arts extracurriculars themselves did not have a direct effect with general academic performance but had indirect effects with visual arts score in the third year (indirect 2: b* = 0.028). These results imply that the participants in the visual arts club were likely to achieve higher visual arts and academic scores. However, whether these indirect effects contribute to the overall effect depends on the sample. Although the total effects were diluted in a sample including children in extracurricular sports, the total effect of extracurricular visual art was significantly positive when comparing extracurricular visual arts, music, and other cultural clubs, or a group of children who do not belong to any club activities. This implies that the effect of visual art learning that transfers to academic performance is evident in the third year, especially when compared with the effect of participation in other cultural clubs and non-participation in clubs.

While the effect size was quite small, like music activities, it suggests that the first and second hypothesis by Winner et al.^8^ could apply to visual arts. The purpose of visual arts education was divided into the development of knowledge and skills in visual arts, and the development of individuals’ general abilities, personality, and identity through artistic activities (e.g., art viewing and creation). Read^33^, a philosopher in arts and arts education, demonstrated that when individuals are provided the instruction of visual arts knowledge and skills, it results in the development of various abilities, including psychological growth as well as that of knowledge and skills. Although this study was not able to show what kind of education the participants received at extracurricular activities, the current finding presumably supports the assumption that development takes place through visual arts activity, as suggested by philosophers.

There are several limitations in this study. First, the current longitudinal survey could not specify what kind of activities in each type of extracurricular activity improve the arts and academic performance. Many experiments have been conducted on the impact of arts education that specify the pathway of cognitive and social developments focusing on detailed instructions in arts education^9,34–36^. Future experiments can contribute to determine the mechanism of transfer and estimate the causal relationship of arts education and general academic performance with higher accuracy.

Second, it should be noted that the impact of extracurricular activity in the arts can differ between race, regional and cultural backgrounds. Especially, cultural background is an important factor in understanding the impact of extracurricular activity in the arts. Some countries provide extracurricular activities in schools, while others do not. Further, the contents, procedure, and amount of time spent in arts education can be different. Future studies should analyze these differences using meta-surveys.

Third, this study collected data from 488 students to examine the relationship between extracurricular art and music activities and academic performance, but the study resulted in an unbalanced design because few students participate in extracurricular activities such as music and art. In the examination of the hypotheses in this study, the transfer effect of extracurricular music and visual arts were different between comparing the children in extracurricular music and visual arts and the other children and comparing children in extracurricular music and visual arts and the other cultural extracurriculars or those who do not participated in extracurriculars. Future research should examine the effects of art and music club activities in an experimental design with a variety of comparisons of balanced numbers of participants. Further, this was a survey study that examined the results of free-will choice of club activities. Therefore, the relationship between participation in club activities and academic performance shown by the results of this study may not only involve more than just participation in club activities but also the choice to do so. In the future, it is necessary to clarify the causal relationship between extracurricular activities related to art and academic performance by constructing a more experimental research design.

## Methods

### Participants

**Fig. 4:**
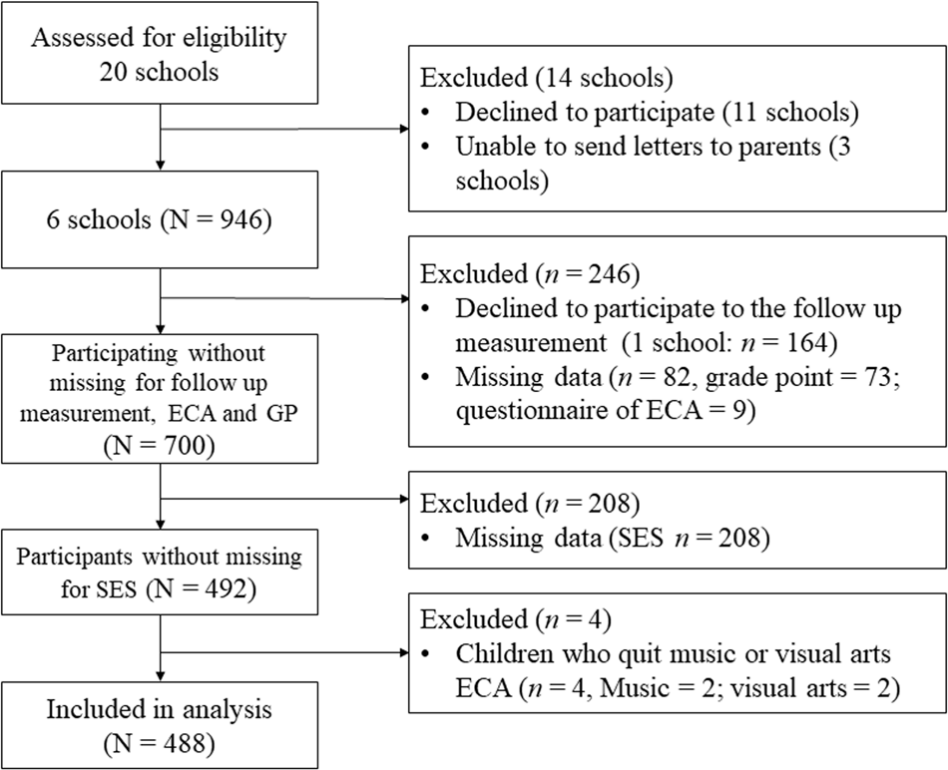
Flow diagram of the recruitment of children and missing data. ECA extracurricular activity.

Figure 4 shows a flow diagram of participant recruitment and the follow-up process. We requested 20 public junior high schools in Sapporo, which is the prefectural capital of Hokkaido in northern Japan, and cities near Sapporo for permission to recruit seventh^-^grade students (i.e., 12–13-year-olds). Eleven schools declined to participate, and three schools declined to send letters to their parents. A total of 946 first-year junior high school students were recruited from six public schools. Students in one school did not participate in the follow-up measurement (*n* = 164), and 82 participants were excluded due to missing data (*n* = 73 for grade points, *n* = 9 for questionnaire of extracurricular activities). Further, excluding SES missing data (*n* = 208), complete data on academic performance, grade points in music and visual arts, and extracurricular activities questionnaire were available for 492 children (males, *n* = 261; females, *n* = 231). After excluding the children who quit music or visual arts extracurriculars, the final data were 488 children (males, *n* = 259; females, *n* = 229). Their participation in extracurricular activities did not last in the follow-up measurement (less than a year). The explanation letter for the present research informed parents/guardians that all data collected from the students and their parents/guardians would be anonymous (i.e., we would not collect personal information such as students’ names and birth dates). Only students whose parents/guardians consented to participation, as evidenced by the return of the questionnaires, were included. Participants and their parents or legal guardians did not provide written informed consent with their signatures for participation in the current study. The study was approved by the institutional review board of the Hokkaido University of Education and the principals of participating schools, and it was conducted in accordance with the approved guidelines. Table 2 shows the characteristics of the participating children.

### Procedure and measures

The academic year runs from April to March in the next year. We collected data on the participants’ academic performance at the end of the first and third years (i.e., two years apart). The participants’ SES was assessed in October when they were in the first year. In the following, ECA means extracurricular activity, and GP means final grade point in a subject summarized at the end of a school year. Further, the music GP, visual arts GP, and general academic performance, which was calculated by five major subjects GP, were abbreviated to MUGP (music GP), VAGP (visual arts GP), and ACGP (academic GP), respectively.

#### Grade point of each art-related subject and general academic performance

In Japan, the government course guidelines determine a common scoring system for all subjects. Teachers rate each students’ development on a three-point scale in each learning unit, based on the following three perspectives: (1) knowledge and techniques; (2) thinking, judgment, and expression; and (3) attitude toward active learning. These ratings are generally scored with a rubric, which includes evaluation criteria established for various situations such as classroom activities, worksheets, remarks, sketches, etc. Thereafter, at the end of the semester or school year, teachers calculate the sum of the rating scores and summarize it into a 5-point scale. The MUGP and VAGP in the national standard curriculum were also obtained from the schools. Each score ranged from 1 to 5 points, with high GPs indicating high performance in each subject. We analyzed an individual’s MUGP and VAGP at the end of the first and third year. As an ACGP, the GPs of five academic subjects (Japanese, mathematics, social studies, science, and English) in the national standard curriculum were obtained from schools. The current study focused on the five core subjects, as they are considered academic performances for high school entrance exams.

#### Socioeconomic status

SES was assessed only as a baseline using a five-point questionnaire administered to participants’ parents and guardians, which questioned their household income (from 2 million yen to 8 million yen), and maternal educational attainment (ranging from completing junior high school to earning a bachelor’s degree). We obtained SES data consisting of household income and maternal educational attainment from 492 participants due to non-respondents.

#### Learning habits

Learning habits were assessed using the duration of learning after school on weekdays and weekends. The participants answered the question of learning duration on weekdays (“How much time do you study on weekdays after school?”) using five categories ((1) more than 3 h; (2) more than 2 h and less than 3 h; (3) more than an hour and less than 2 h; (4) more than 30 min and less than an hour; and (5) less than 30 min). In addition, they were also asked, “How much time do you study in a day on weekends?” using the five categories described above.

#### Extracurricular activity

The participants completed a questionnaire regarding their participation in sports and cultural activities during October while in the first and third years. They were asked a single question (“What extracurricular activity are you involved in?”), and their responses were coded as follows: (1) I belong to sports activity; (2) I belong to cultural activity; and (3) I do not belong to any activity. Further, when participants checked (2) but did not participate in brass band, choir, and fine arts, the ECA culture was coded as 1 and the others as 0. The ECA culture demonstrates whether the participants were involved in cultural clubs other than visual art and music, such as computer, cooking, and broadcasting clubs.

We defined the ECA music and visual arts with the following definition. Participants in the brass band and choir (*N* = 55; males = 8, females = 47) were coded as participants in the music extracurricular activity, with ECA music as 1. In addition, the participants in the visual arts (*N* = 23; males = 5, females = 18) were coded as participants in the visual art extracurricular activity, with ECA visual arts as 1. There was a gender bias, with 80% of the music and visual arts club being female students, which is consistent with the results of a large survey of Japanese junior high school students (Japan Sports Agency, 2018).

This study was not preregistered. The data and study materials of this study are available from the authors.

### Statistical analysis

Structural equation modeling with full-information maximum likelihood estimation was conducted to examine the hypothetical model (Fig. 1) after controlling for household income, maternal education, sex and learning habits. The variables in the experimental and controlled variables were included as explanatory variables for ACGP. When defining ACGP, the ACGP in the first and third years were modeled as the latent variables, which were defined by GPs of five subjects (i.e., Japanese, Social Studies, Math, Science, and English) at each time point. As the current study design did not allow for the accumulation of subject scores through GPA, changes in the final grades in each subject for each grade level were considered. In aggregating ACGP, the total scores in major subjects are likely to reflect high or low scores in specific subjects. Therefore, by latent factorization, we assumed a common factor derived from the five subjects and examined changes in that factor. In addition, learning habits in the first and third years were also modeled as latent variables, which were defined by learning times on weekdays and weekends. The ACGP, MUGP, and VAGP in third year were modeled as being explained by each variable in the first year. The variables in the hypothesis and all the control variables were first included as explanatory variables of objective variables such as ACGP, MUGP, and VAGP. Thereafter, the model was refined by excluding insignificant relationships between the experimental and control variables (other than the main paths related to ECA music and visual arts and SES) and adding covariate relationships between each main subject score and learning habits when the modification indices (the change of *χ*^2^ value with one degree of freedom when a particular path was added) were more than 3.84. This indicates that the *p* value for the added parameter would be less than .05. The following fit indices for the hypothetical models were calculated: (a) comparative fit index (CFI; should be higher than .95 for good fit), (b) goodness of fit index (GFI; should be higher than .95 for good fit), (c) adjusted goodness of fit index (AGFI; should be higher than .95 for good fit), (d) RMSEA (should be .08 or lower), and (e) standardized root-mean residual (SRMR; should be .05 or lower)^37^. All statistical analyses were conducted using R, version 4.1.0 and psych package^38^ for descriptive statistics and lavaan package 0.6–11^39^ for structural equation modeling and ggplot2^40^ for visualization. The R analysis code before adding covariance and excluding non-significant paths is described in Supplementary Method.

### Reporting summary

Further information on research design is available in the Nature Research Reporting Summary linked to this article.

## Supplementary information

Supplementary Information

Reporting Summary

## Data Availability

The datasets analyzed during the current study are not publicly available as their containing information could compromise the privacy of research participants but are available from the corresponding author on reasonable request.

## Code availability

R scripts used for the current study are available in Supplementary Method.
