# Supplementary material for: Extracurricular music and visual arts activities are related to academic performance improvement in school-aged children
Source: NPJ Sci Learn. 2023 Mar 29;8:7. doi: 10.1038/s41539-023-00155-0 (PMC10060367; doi:10.1038/s41539-023-00155-0)
Supplement: Supplementary file 1 — Supplementary Information [file 41539_2023_155_MOESM1_ESM.pdf]

Supplementary information

Supplementary Table 1. Estimates and standardized effects of music and visual arts ECA and control variables on academic performance

		Estimate	SE	Z	P	Lower	Upper	Stad.all
ACGP (3rd year)	~							
ACGP (1st year)		0.777	0.045	17.414	0.000	0.689	0.864	0.702
ECA Visual Art		0.059	0.080	0.742	0.458	-0.097	0.216	0.018
ECA Music		-0.126	0.055	-2.297	0.022	-0.234	-0.019	-0.058
MUGP (3rd year)		0.176	0.025	6.988	0.000	0.127	0.226	0.229
VAGP (3rd year)		0.171	0.025	6.849	0.000	0.122	0.219	0.207
sex		0.094	0.036	2.634	0.008	0.024	0.163	0.069
Learning habits (3rd year)		-0.075	0.021	-3.634	0.000	-0.116	-0.035	-0.117
MUGP (3rd year)	~							
MUGP (1st year)		0.523	0.046	11.261	0.000	0.432	0.614	0.443
ECA Music		0.401	0.107	3.763	0.000	0.192	0.610	0.142
Learning habits (3rd year)		-0.229	0.042	-5.502	0.000	-0.311	-0.148	-0.276
VAGP (3rd year)	~							
VAGP (1st year)		0.388	0.038	10.139	0.000	0.313	0.463	0.408
ECA Visual Art		0.356	0.154	2.312	0.021	0.054	0.658	0.088
sex		-0.313	0.065	-4.816	0.000	-0.441	-0.186	-0.190
Maternal education		0.126	0.032	3.984	0.000	0.064	0.187	0.153
Learning habits (3rd year)		-0.071	0.037	-1.903	0.057	-0.144	0.002	-0.091
ACGP (1st year)	~							
ECA Visual Art		-0.031	0.104	-0.294	0.768	-0.235	0.173	-0.010
ECA Music		-0.250	0.070	-3.569	0.000	-0.387	-0.113	-0.127
MUGP (1st year)		0.387	0.035	10.959	0.000	0.318	0.456	0.471
VAGP (1st year)		0.228	0.028	8.188	0.000	0.174	0.283	0.322
sex		0.118	0.047	2.521	0.012	0.026	0.209	0.096
Household income		0.066	0.021	3.134	0.002	0.025	0.107	0.112
Maternal education		0.086	0.021	4.023	0.000	0.044	0.127	0.140
MUGP (1st year)	~							
ECA Music		0.281	0.094	2.983	0.003	0.096	0.466	0.118

sex	-0.445	0.063	-7.076	0.000	-0.568	-0.322	-0.298
Household income	0.169	0.030	5.624	0.000	0.110	0.228	0.235
Maternal education	0.063	0.031	2.029	0.042	0.002	0.125	0.085
Learning habits (1st year)	-2.565	1.166	-2.199	0.028	-4.850	-0.279	-0.546
VAGP (1st year) ~							
ECA Visual Art	0.486	0.172	2.828	0.005	0.149	0.823	0.114
sex	-0.419	0.075	-5.616	0.000	-0.565	-0.273	-0.242
Household income	0.101	0.036	2.763	0.006	0.029	0.172	0.121
Maternal education	0.066	0.038	1.724	0.085	-0.009	0.140	0.076
Learning habits (1st year)	-3.149	1.549	-2.032	0.042	-6.185	-0.112	-0.578

Note: ECA: extracurricular activity, MUGP: music GP, VAGP: visual arts GP, ACGP: general academic score which is calculated by five major subjects GP.

Supplementary Table 2. Standardized direct, indirect, and total effects for all variables in the model excluding data of children who joined extracurricular sports clubs (N=147)

	Estimate	SE	Z	P	Lower	Upper	Stad.all
Music							
direct	-0.017	0.069	-0.245	0.807	-0.152	0.118	-0.012
indirect on ACGP in first year via MUGP in first year (indirect 1)	0.136	0.051	2.649	0.008	0.035	0.236	0.102
total on ACGP in first year from ECA	-0.073	0.106	-0.694	0.488	-0.280	0.134	-0.055
total on MUGP in third year via MUGP in first year	0.708	0.138	5.122	0.000	0.437	0.979	0.358
indirect on ACGP in third year via MUGP in third year (indirect 2)	0.143	0.043	3.307	0.001	0.058	0.228	0.105
total on ACGP in third year from ECA	0.075	0.102	0.728	0.466	-0.126	0.275	0.055
Visual Arts							
direct	0.192	0.089	2.159	0.031	0.018	0.367	0.102
indirect on ACGP in first year via VAGPin first year (indirect 1)	0.072	0.054	1.317	0.188	-0.035	0.179	0.039
total on ACGP in first year from ECA	0.110	0.145	0.758	0.449	-0.174	0.393	0.060
total on VAGP in third year via VAGP in first year	0.538	0.189	2.840	0.005	0.167	0.909	0.219
indirect on ACGP in third year via VAGP in third year (indirect 2)	0.048	0.029	1.667	0.096	-0.009	0.105	0.026
total on ACGP in third year from ECA	0.318	0.132	2.407	0.016	0.059	0.577	0.168

Note: GP: final grade point in a subject summarized at the end of a school year, MUGP: music GP, VAGP: visual arts GP, ACGP: general academic score which is calculated by five major subjects GP.

Supplementary Table 3. Estimates and standardized effects of music and visual arts ECA and control variables on academic performance excluding data of children who joined extracurricular sports clubs (N=147)

		Estimate	SE	Z	P	Lower	Upper	Stad.all
ACGP (3rd year)	~							
ACGP (1st year)		0.702	0.074	9.498	0.000	0.557	0.847	0.682
ECA Visual Art		0.192	0.089	2.159	0.031	0.018	0.367	0.102
ECA Music		-0.017	0.069	-0.245	0.807	-0.152	0.118	-0.012
MUGP (3rd year)		0.202	0.046	4.379	0.000	0.112	0.292	0.293
VAGP (3rd year)		0.090	0.044	2.065	0.039	0.005	0.175	0.117
sex		0.135	0.067	2.014	0.044	0.004	0.266	0.094
Learning habits (3rd year)		-0.119	0.035	-3.393	0.001	-0.188	-0.050	-0.203
MUGP (3rd year)	~							
MUGP (1st year)		0.546	0.078	6.982	0.000	0.392	0.699	0.471
ECA Music		0.507	0.126	4.016	0.000	0.260	0.755	0.256
Learning habits (3rd year)		-0.214	0.066	-3.249	0.001	-0.344	-0.085	-0.251
VAGP (3rd year)	~							
VAGP (1st year)		0.532	0.066	8.018	0.000	0.402	0.662	0.529
ECA Visual Art		0.396	0.160	2.475	0.013	0.082	0.709	0.161
sex		-0.144	0.125	-1.153	0.249	-0.388	0.101	-0.077
Maternal education		0.050	0.056	0.889	0.374	-0.060	0.160	0.058
Learning habits (3rd year)		-0.151	0.056	-2.701	0.007	-0.261	-0.042	-0.197
ACGP (1st year)	~							
ECA Visual Art		0.038	0.137	0.275	0.784	-0.232	0.307	0.021
ECA Music		-0.209	0.096	-2.188	0.029	-0.396	-0.022	-0.158
MUGP (1st year)		0.369	0.070	5.285	0.000	0.232	0.506	0.475
VAGP (1st year)		0.269	0.060	4.503	0.000	0.152	0.386	0.358
sex		-0.064	0.103	-0.618	0.537	-0.267	0.139	-0.046
Household income		0.050	0.042	1.200	0.230	-0.032	0.133	0.088
Maternal education		0.056	0.044	1.272	0.203	-0.030	0.143	0.087
MUGP (1st year)	~							
ECA Music		0.368	0.120	3.059	0.002	0.132	0.604	0.215
sex		-0.573	0.125	-4.603	0.000	-0.817	-0.329	-0.318
Household income		0.178	0.053	3.327	0.001	0.073	0.282	0.240
Maternal education		-0.026	0.059	-0.437	0.662	-0.142	0.090	-0.031

Learning habits (1st year)	-0.269	0.063	-4.291	0.000	-0.392	-0.146	-0.331
VAGP (1st year) ~							
ECA Visual Art	0.267	0.194	1.377	0.169	-0.113	0.647	0.109
sex	-0.431	0.146	-2.959	0.003	-0.716	-0.145	-0.231
Household income	0.148	0.063	2.359	0.018	0.025	0.271	0.193
Maternal education	0.001	0.071	0.011	0.991	-0.139	0.140	0.001
Learning habits (1st year)	-0.101	0.072	-1.408	0.159	-0.242	0.040	-0.120

Note: ECA: extracurricular activity, MUGP: music GP, VAGP: visual arts GP, ACGP: general academic score which is calculated by five major subjects GP.

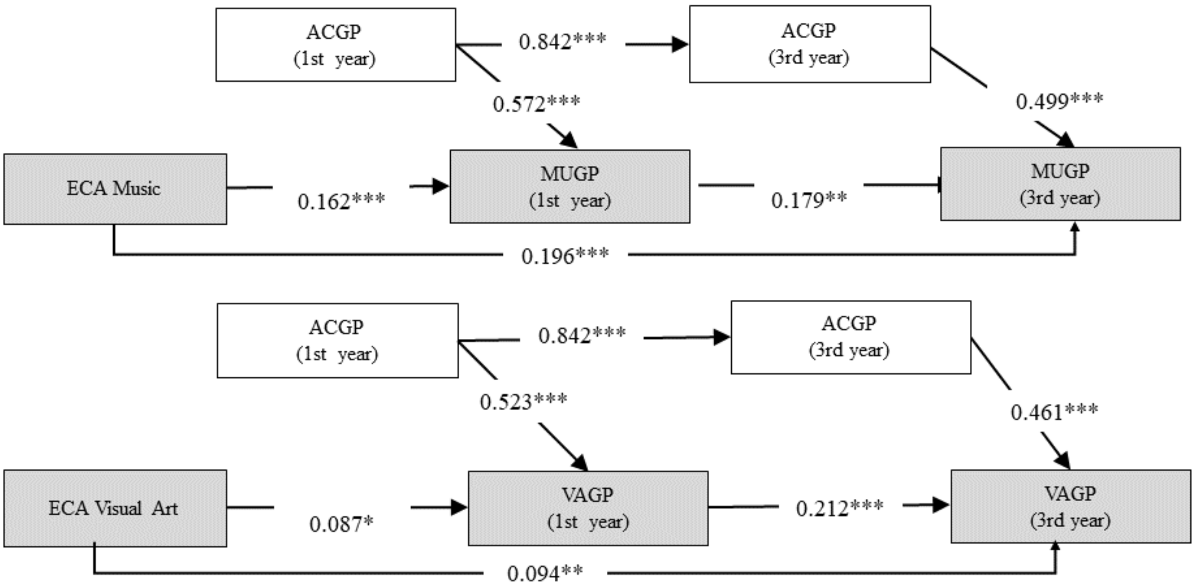

Supplementary Figure 1. Direct and indirect pathways from extracurricular activity in music and visual arts to GP of art-related subjects, mediated by academic performance scores with SES controls.

Note: Structural equation modeling N = 488, χ^2 (df = 167) = 350.58 (p < .001), CFI = 0.97, GFI = 0.99, AGFI = 0.98, RMSEA = 0.05; dotted paths denote non-significant effects, while solid-line paths denote significant effects. Values are presented as standardized $\beta(b^*)$, after controlling for sex, household income, maternal education and learning habits. ECA: extracurricular activity, GP: final grade point in a subject summarized at the end of a school year, MUGP: music GP, VAGP: visual arts GP, ACGP: general academic score which is calculated by five major subjects GP.

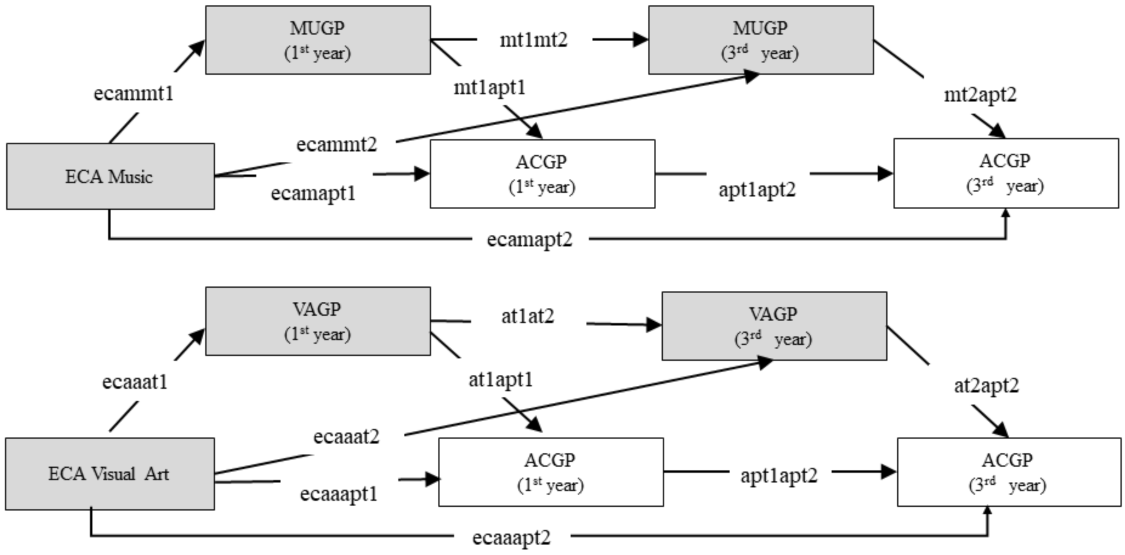

Supplementary Figure 2. Modeling schema depicting with R objects for analyzing the direct and indirect pathways from extracurricular activity in music and visual arts to GP of art-related subjects. Each abbreviation is shown in Supplementary Methods.

Supplementary Method

```
library(lavaan)
model<-"
ACGP_T1 = ~ Japanese_T1 + SocialStudies_T1 + Mathematics_T1 + Science_T1 + English_T1
ACGP_T2 = ~ Japanese_T2 + SocialStudies_T2 + Mathematics_T2 + Science_T2 + English_T2
LearningHabits_T1 = ~ LearningHabits_weekday_T1 + LearningHabits_weekend_T1
LearningHabits_T2 = ~ LearningHabits_weekday_T2 + LearningHabits_weekend_T2
ACGP_T2 ~ apt1apt2*ACGP_T1 + ecaaapt2*ECA_Art + ecamapt2*ECA_Music + mt2apt2* MUGP _T2
  + at2apt2* VAGP _T2 + sex + Income + Maternal + Learning_T2
MUGP_T2 ~ mt1mt2*MUGP_T1 + ecammt2*ECA_Music + sex + Income + Maternal + Learning_T2
VAGP _T2 ~ at1at2* VAGP _T1 + ecaaat2*ECA_Art + sex + Income + Maternal + Learning_T2
ACGP_T1 ~ ecaaapt1*ECA_Art + ecamapt1*ECA_Music + mt1apt1*MUGP_T1 + at1apt1* VAGP _T1 +
sex + Income + Maternal + Learning_T1
MUGP_T1 ~ ecammt1*ECA_Music + sex + Income + Maternal + Learning_T1
VAGP _T1 ~ ecaaat1*ECA_Art + sex + Income + Maternal + Learning_T1

#indirect ACGP_T1 via each subject
music_ind_apt1 := ecammt1*mt1apt1
art_ind_apt1 := ecaaat1*at1apt1

#total ACGP_T1
music_total_apt1 := ecammt1*mt1apt1+ecamapt1
art_total_apt1 := ecaaat1*at1apt1+ecaaapt1

#total each subject T2
music_total_mt2 := ecammt1*mt1mt2+ecammt2
art_total_at2 := ecaaat1*at1at2+ecaaat2

#indirect ACGP_T2 via each subject at T2
music_ind_apt2 := (ecammt1*mt1mt2+ecammt2)*mt2apt2
art_ind_apt2 := (ecaaat1*at1at2+ecaaat2)*at2apt2

#total ACGP_T2
music_total_apt2:=ecamapt2+((ecammt1*mt1mt2+ecammt2)*mt2apt2)+(ecammt1*mt1apt1+ecamapt1)*a
pt1apt2
art_total_apt2:=ecaaapt2+((ecaaat1*at1at2+ecaaat2)*at2apt2)+(ecaaat1*at1apt1+ecaaapt1)*apt1apt2
```

```
"
result <- sem(model, data = data, fixed.x = FALSE, missing="fiml")
fitMeasures(result)
summary(result, fit.measures = TRUE, modindices = FALSE, ci = TRUE,standardized=TRUE)
```

Note: T1 means the first year and T2 means the third year. ACGP, MUGP, and VAGP mean general academic performance, music score, and visual arts score, respectively. The constants assigned to the path coefficients are shown in the Supplementary Figure 2.
