# Supplementary material for: Extracurricular music and visual arts activities are related to academic performance improvement in school-aged children
Source: NPJ Sci Learn. 2023 Mar 29;8:7. doi: 10.1038/s41539-023-00155-0 (PMC10060367; doi:10.1038/s41539-023-00155-0)
Supplement: Supplementary file 2 — Reporting Summary [file 41539_2023_155_MOESM2_ESM.pdf]

nature portfolio

Corresponding author(s): Noriteru Morita
Last updated by author(s): Jun 7, 2022

Reporting Summary

Nature Portfolio wishes to improve the reproducibility of the work that we publish. This form provides structure for consistency and transparency in reporting. For further information on Nature Portfolio policies, see our Editorial Policies and the Editorial Policy Checklist.

Statistics

For all statistical analyses, confirm that the following items are present in the figure legend, table legend, main text, or Methods section.

n/a	Confirmed	
☐	☒	The exact sample size (*n*) for each experimental group/condition, given as a discrete number and unit of measurement
☐	☒	A statement on whether measurements were taken from distinct samples or whether the same sample was measured repeatedly
☐	☒	The statistical test(s) used AND whether they are one- or two-sided<br>*Only common tests should be described solely by name; describe more complex techniques in the Methods section.*
☐	☒	A description of all covariates tested
☐	☒	A description of any assumptions or corrections, such as tests of normality and adjustment for multiple comparisons
☐	☒	A full description of the statistical parameters including central tendency (e.g. means) or other basic estimates (e.g. regression coefficient) AND variation (e.g. standard deviation) or associated estimates of uncertainty (e.g. confidence intervals)
☐	☒	For null hypothesis testing, the test statistic (e.g. *F*, *t*, *r*) with confidence intervals, effect sizes, degrees of freedom and *P* value noted<br>*Give P values as exact values whenever suitable.*
☒	☐	For Bayesian analysis, information on the choice of priors and Markov chain Monte Carlo settings
☒	☐	For hierarchical and complex designs, identification of the appropriate level for tests and full reporting of outcomes
☐	☒	Estimates of effect sizes (e.g. Cohen's *d*, Pearson's *r*), indicating how they were calculated

Our web collection on statistics for biologists contains articles on many of the points above.

Software and code

Policy information about availability of computer code

Data collection	No software was used for data collection.
Data analysis	All statistical analyses were conducted using R, version 4.1.0 and psych package (Revelle, 2021) for descriptive statistics and lavaan package 0.6-11 (Rosseel, 2012) for structural equation modeling and ggplot2 (Wickham, 2016) for visualization. The R analysis code before adding covariance and excluding non-significant paths is described in Supplementary R code.

For manuscripts utilizing custom algorithms or software that are central to the research but not yet described in published literature, software must be made available to editors and reviewers. We strongly encourage code deposition in a community repository (e.g. GitHub). See the Nature Portfolio guidelines for submitting code & software for further information.

Data

Policy information about availability of data

All manuscripts must include a data availability statement. This statement should provide the following information, where applicable:

- Accession codes, unique identifiers, or web links for publicly available datasets
- A description of any restrictions on data availability
- For clinical datasets or third party data, please ensure that the statement adheres to our policy

The datasets generated during and/or analysed during the current study are available from the corresponding author on reasonable request.

Human research participants

Policy information about studies involving human research participants and Sex and Gender in Research.

Reporting on sex and gender	In this manuscript, we used the term "sex" because we obtained the data from school records based on biological differences between males and females. And, our results are shown after controlling for sex differences.
Population characteristics	See above.
Recruitment	See above.
Ethics oversight	The ethical review board of the Hokkaido University of Education.

Note that full information on the approval of the study protocol must also be provided in the manuscript.

Field-specific reporting

Please select the one below that is the best fit for your research. If you are not sure, read the appropriate sections before making your selection.

☐ Life sciences ☒ Behavioural & social sciences ☐ Ecological, evolutionary & environmental sciences

For a reference copy of the document with all sections, see nature.com/documents/nr-reporting-summary-flat.pdf

Behavioural & social sciences study design

All studies must disclose on these points even when the disclosure is negative.

Study description	This study was performed using a 2-year longitudinal observational study design and analyzed quantitative and qualitative data.
Research sample	A total of 488 (boys/girls = 259/229) students aged 12-13 years, who lived in Hokkaido, Japan, participated in this study. These students were from five public schools in different cities in Hokkaido, Japan.
Sampling strategy	Recruitment of participants was performed as follows: (1) 20 public junior high schools with 3–6 classes per grade were asked to participate in this study, (2) 14 schools declined participation, and (3) a school was lost to the 2-year follow-up. Finally, 488 children were followed. Data collected from these students were then analyzed.<br>Power analyses with post-hoc procedure were conducted to determine statistical power using G*Power 3.1.2. The effect size was defined as small, medium, and large when Cohen's f2 = .02, .15, and .35, respectively.
Data collection	The grade point of each academic subject, as a measure of academic performance, was evaluated by teachers of each academic subject throughout a school year, based on class activities, and reflection papers, and paper test scores. Questionnaires for daily lifestyle and family's socioeconomic status were answered by children themselves and their parents/guardians, respectively (see the Methods section).
Timing	Baseline data for body size, physical fitness, and lifestyle were assessed in May, June, and October 2012, respectively. Grade points of academic subjects in the 7th grade were determined at the end of the school year, i.e., March 2013 (in Japan, the school year starts in April and ends in March of the following year). Follow-up data for body size, physical fitness, lifestyle, and grade points were collected in the same months after 2 years (May 2014 to March 2015).
Data exclusions	No data were excluded from this study except for children who lost data for grade points.
Non-participation	One school was dropped out at follow-up measurement because of missing data.
Randomization	None.

Reporting for specific materials, systems and methods

We require information from authors about some types of materials, experimental systems and methods used in many studies. Here, indicate whether each material, system or method listed is relevant to your study. If you are not sure if a list item applies to your research, read the appropriate section before selecting a response.

Materials & experimental systems

n/a	Involved in the study	
☒	☐	Antibodies
☒	☐	Eukaryotic cell lines
☒	☐	Palaeontology and archaeology
☒	☐	Animals and other organisms
☒	☐	Clinical data
☒	☐	Dual use research of concern

Methods

n/a	Involved in the study	
☒	☐	ChIP-seq
☒	☐	Flow cytometry
☒	☐	MRI-based neuroimaging
